# Epigastric typhoid ileal perforation unmasking an incidental adult midgut malrotation: a rare clinical co-existence

**DOI:** 10.1093/jscr/rjag861

**Published:** 2026-09-30

**Authors:** Hafiz Syed Zaigham Ali Shah, Abdul Wahab Haider, Ayesha Mahmood, Fatima Syed, Syed Saqib Ali Shah, Jamal Anwar

**Affiliations:** General Surgery Department, Aberdeen Royal Infirmary/University of Aberdeen, King's Street, Aberdeen AB24 3FX, United Kingdom; General Surgery Department, King Edward Medical University/Mayo Hospital, Nila Gumbad Road, Lahore 5400, Punjab, Pakistan; King Edward Medical University/Mayo Hospital, Nila Gumbad Road, Lahore 5400, Punjab, Pakistan; King Edward Medical University/Mayo Hospital, Nila Gumbad Road, Lahore 5400, Punjab, Pakistan; Aberdeen Royal Infirmary/University of Aberdeen, King's Street, Aberdeen AB24 3FX, United Kingdom; Combined Military Hospital, Lahore/Medical College and Institute of Dentistry, Lahore 5400, Punjab, Pakistan; Rawalpindi Medical University, Rawalpindi 4600, Punjab, Pakistan; King Edward Medical University/Mayo Hospital, Nila Gumbad Road, Lahore 5400, Punjab, Pakistan

**Keywords:** typhoid ileal perforation, adult midgut malrotation, acute abdomen, loop ileostomy

## Abstract

Peritonitis due to typhoid ileal perforation is a life-threatening surgical emergency that remains common in endemic regions but is rarely encountered in developed countries. Anatomically, its presentation is mostly localized to pain in right iliac fossa. A 33-year-old man presented with six days of fever, severe epigastric pain, vomiting, and septic shock. Because of the marked epigastric tenderness and pneumoperitoneum on X-rays, perforated peptic ulcer was the leading preoperative diagnosis. Emergency laparotomy instead revealed severe peritoneal contamination, incidental incomplete midgut malrotation with the caecum and ileocaecal junction in the epigastrium, and a 2 × 2 cm ileal perforation 15 cm proximal to the ileocaecal junction. A loop ileostomy was performed. Histology and blood cultures confirmed typhoid fever. The patient recovered uneventfully and underwent successful ileostomy reversal. This case highlights how intestinal malrotation can disguise the classical presentation of typhoid perforation, emphasizing the importance of diagnostic suspicion and intraoperative adaptability.

## Introduction

Typhoid fever, caused by *Salmonella typhi*, remains one of the significant causes of morbidity in South Asia, sub-Saharan Africa, and Southeast Asia, with an estimated 11–21 million cases reported annually [1]. Ileal perforation complicates roughly 0.8%–3% of hospitalized cases and has a mortality of ~30% even with prompt surgery [2]. Surgeons outside these countries having better healthcare systems will encounter this occasionally in diaspora population. The presentation is usually predictable which fever is for 1 to 2 weeks, then right iliac fossa peritonism reflecting where the terminal ileum normally sits.

Adult midgut malrotation is a congenital failure of normal 270° counterclockwise gut rotation during fetal development [3]. This condition is mostly asymptomatic. Prevalence is ~0.2% [4], and the condition is typically diagnosed only when something else brings the patient to theatre.

We describe a case where these two conditions collided, producing a presentation that neither of us had encountered before and that the operating surgeon was not prepared for until the abdomen was open.

## Case report

A 33-year-old man was referred to the emergency department from a peripheral facility after 6 days of high-grade fever and 4 days of worsening epigastric and right upper quadrant pain. He had not been vaccinated against typhoid, denied recent travel, and had no prior abdominal surgery or history of inflammatory bowel disease and tuberculosis. He was commenced on oral antibiotics peripherally but did not show any improvement.

On arrival, he was in septic shock with a blood pressure of 88/56 mmHg, heart rate 118 bpm, temperature 38.9°C. The abdomen was board-rigid, peritonism worst in the epigastrium and right upper quadrant. Bloods showed leucocytosis 17.9 × 10^9^/L and C-reactive proteins 324 mg/L. Erect chest X-ray (CXR) confirmed bilateral free subdiaphragmatic air (Fig. 1). given haemodynamic instability and unequivocal perforation peritonitis, we proceeded directly to emergency midline laparotomy without CT abdomen and pelvis.

**Figure 1:**
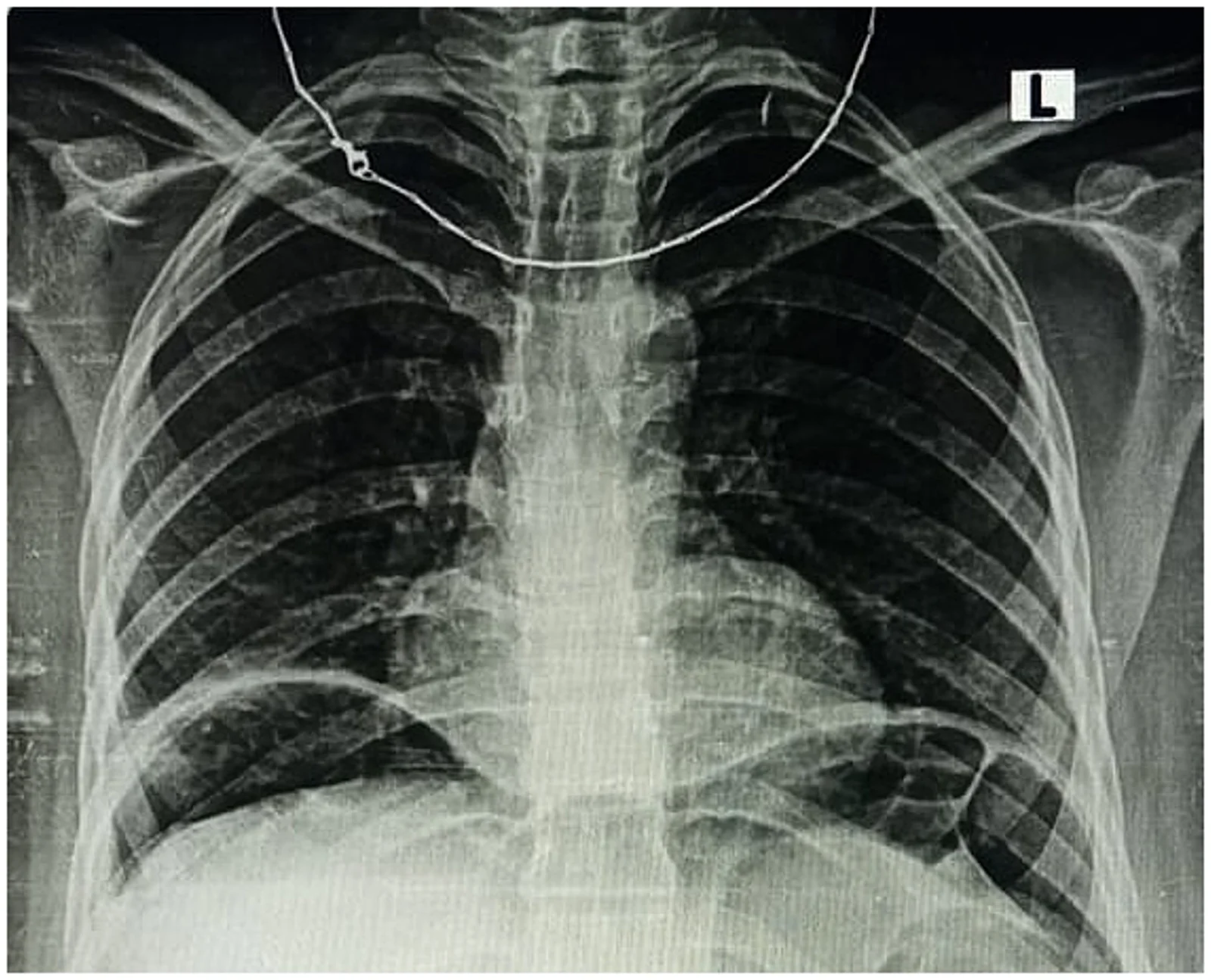
Erect CXR - bilateral free sub diaphragmatic air.

On exploratory laparotomy, we encountered roughly 2 L of turbid, bile-stained fluid. What stopped us was the anatomy. The caecum and ileocaecal junction were located in the epigastrium rather than the right iliac fossa. The entire small bowel occupied the right side of the abdomen, while the colon was positioned on the left (Fig. 2). the small bowel mesentery had a narrow base, consistent with incomplete intestinal malrotation. There were no Ladd’s bands. This was an unsuspected incomplete midgut malrotation.

**Figure 2:**
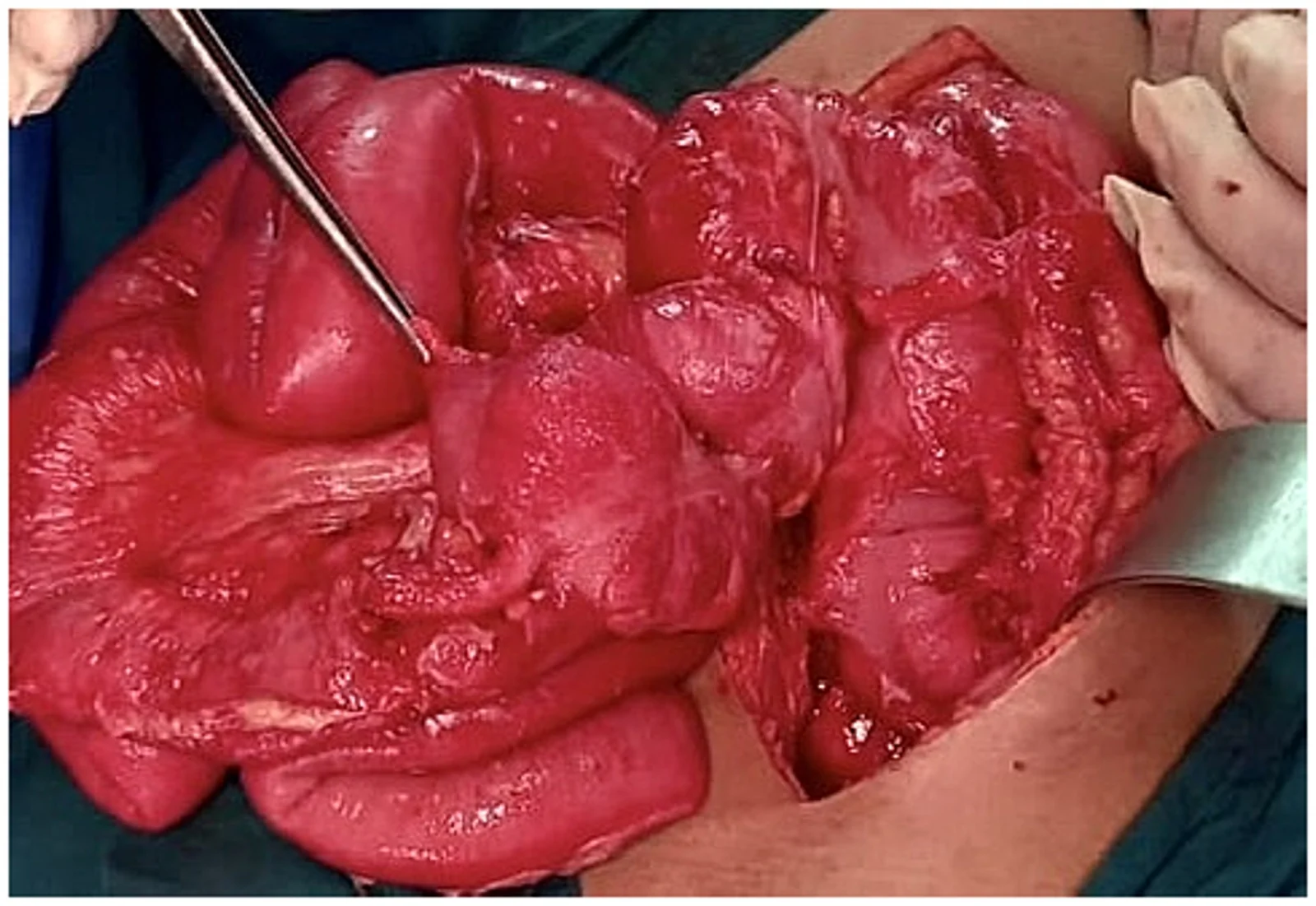
Intraoperative picture of colon - malrotation of gut.

Working proximally from the displaced ileocaecal junction, we found a single 2 × 2 cm perforation 15 cm away with thickened, oedematous, and friable edges, surrounded by fibrinous exudate. Primary repair or resection with anastomosis was deemed unsafe option given the degree of contamination and the patient’s unstable physiological state. We fashioned a loop ileostomy, irrigated thoroughly, and placed a pelvic drain.

He was stepped down from High Dependency Unit by Day 3 and went home on Day 9. Blood cultures confirmed *Salmonella typhi*; a 6-week antibiotic course was completed. Histology showed mucosal ulceration with acute inflammatory infiltrate and fibrinous peritonitis but no granulomata or dysplasia, consistent with typhoid ulceration. The patient returned 3 months later for elective ileostomy reversal. A preoperative loopogram Contrast Study through Ileostomy Loop confirmed the abnormal orientation of the midgut, demonstrating the entire colon on the left side (Fig. 3), consistent with the intraoperative findings of incomplete intestinal malrotation. The ileostomy reversal was performed uneventfully, and the patient remained well on subsequent follow-up.

**Figure 3:**
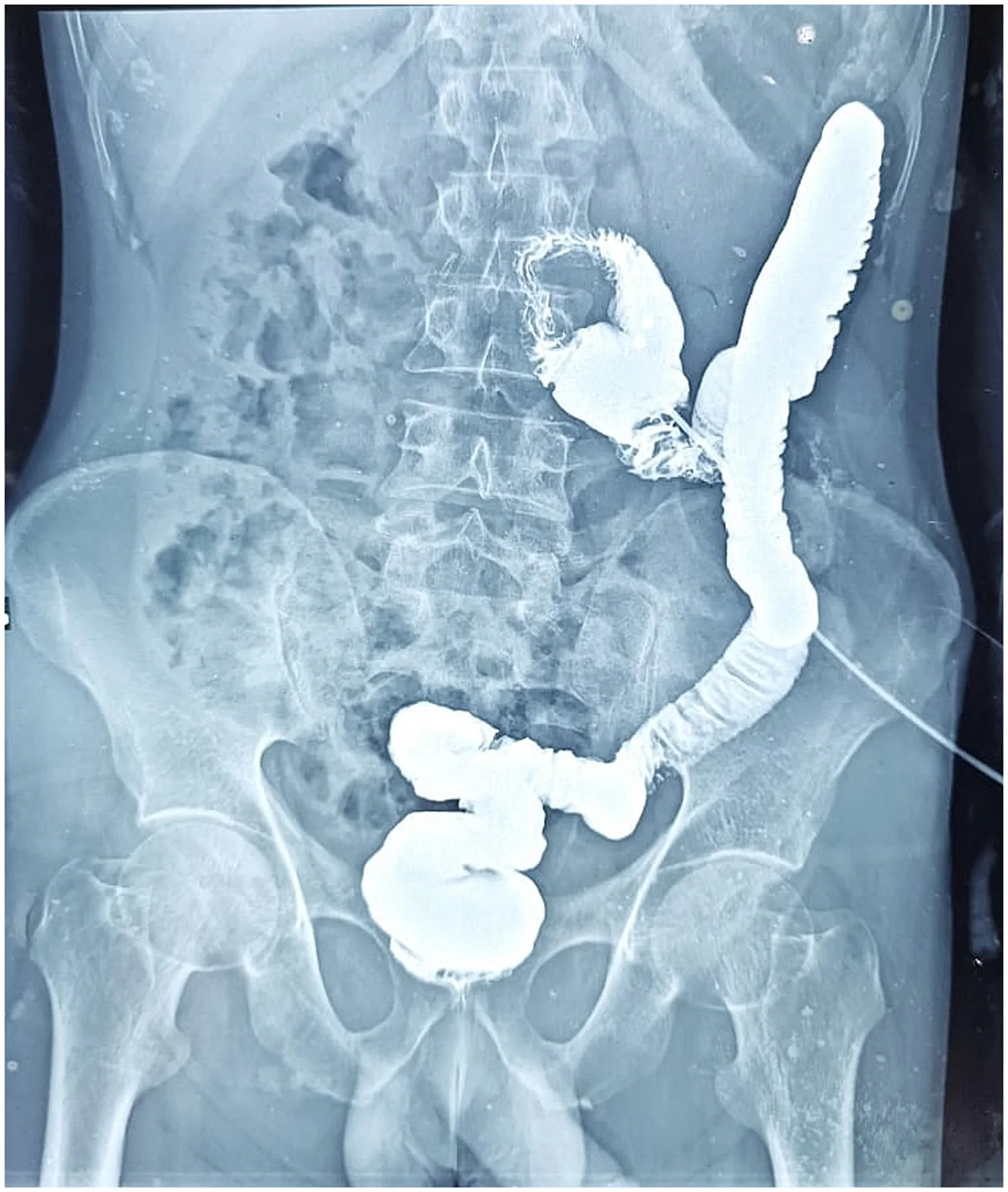
Post-operative contrast loopography demonstrated colon anatomy - abnormal orientation of the gut.

## Discussion

Marked epigastric peritonism with free gas pointed squarely towards perforated peptic ulcer. Before laparotomy, typhoid perforation was not our top differential. The diagnosis only became clear during the laparotomy.

Typhoid perforation occurs through transmural necrosis of Peyer’s patches in the terminal ileum, typically in the second or third week of untreated infection [5]. In normal anatomy, this produces right-sided peritonism. Here, the malrotation had repositioned the ileocaecal junction and the distal ileum into the epigastrium, shifting both the perforation and the peritonism with it.

It is worth noting that Ladd’s bands, classically described as the hallmark of malrotation, were absent. This is not unusual in incomplete variants [6]. A surgeon unfamiliar with this can be caught off guard, as we were.

On operative strategy, there is no single correct approach to typhoid ileal perforation [7]. Primary repair is justified in clean cases with a stable patient and healthy bowel, conditions that did not apply here. A staged approach with loop ileostomy, supported by the literature in physiologically compromised patients [8], was the right decision. Widal testing was not performed as its poor sensitivity and specificity make it unreliable for operative decision-making [9].

Contrast-enhanced CT scan of abdomen and pelvis (CTAP) is the imaging modality of choice for diagnosing gastrointestinal perforation and can identify intestinal malrotation by demonstrating an abnormally positioned caecum, right-sided small bowel, left-sided colon, and a narrow mesenteric root. However, our patient presented with generalized peritonitis and septic shock, requiring immediate resuscitation and emergency laparotomy without further imaging. This is consistent with current emergency surgery guidelines, which recommend urgent operative management rather than delaying treatment for CTAP in hemodynamically unstable patients [10].

Histology was important beyond confirming the diagnosis. The absence of granulomata effectively excluded Crohn’s disease and intestinal tuberculosis, both of which can mimic typhoid perforation at operation and would have changed long-term management substantially [11].

The broader lesson is about intraoperative adaptability. When the anatomy does not match expectations, the answer is to work with what is in front of us, and not spend time reconciling the unexpected with a fixed pre-operative plan.

## Conclusion

Midgut malrotation displaced the terminal ileum into the epigastrium, resulting in an atypical presentation of typhoid ileal perforation. The diagnosis was made at laparotomy, and a loop ileostomy with staged reversal led to full recovery. This case highlights that atypical peritonism does not exclude typhoid perforation and emphasizes the importance of intraoperative adaptability.
